# CoExp: A Web Tool for the Exploitation of Co-expression Networks

**DOI:** 10.3389/fgene.2021.630187

**Published:** 2021-02-24

**Authors:** Sonia García-Ruiz, Ana L. Gil-Martínez, Alejandro Cisterna, Federico Jurado-Ruiz, Regina H. Reynolds, NABEC (North America Brain Expression Consortium), Mark R. Cookson, John Hardy, Mina Ryten, Juan A. Botía

**Affiliations:** ^1^Institute of Neurology, University College London, London, United Kingdom; ^2^NIHR Great Ormond Street Hospital Biomedical Research Centre, University College London, London, United Kingdom; ^3^Department of Genetics and Genomic Medicine, Great Ormond Street Institute of Child Health, University College London, London, United Kingdom; ^4^Departamento de Ingeniería de la Información y las Comunicaciones, Universidad de Murcia, Murcia, Spain; ^5^Laboratory of Neurogenetics, National Institute on Aging, National Institutes of Health, Bethesda, MD, United States; ^6^Department of Neurodegenerative Diseases, UCL Queen Square Institute of Neurology, London, United Kingdom; ^7^Department of Neurodegenerative Disease, United Kingdom Dementia Research Institute at UCL, UCL Institute of Neurology, University College London, London, United Kingdom; ^8^Reta Lila Weston Institute, UCL Queen Square Institute of Neurology, London, United Kingdom; ^9^UCL Movement Disorders Centre, University College London, London, United Kingdom; ^10^Institute for Advanced Study, The Hong Kong University of Science and Technology, Hong Kong, China

**Keywords:** co-expression network, guilt by association, web app for neuroscience, transcriptomics, brain

## Abstract

Gene co-expression networks are a powerful type of analysis to construct gene groupings based on transcriptomic profiling. Co-expression networks make it possible to discover modules of genes whose mRNA levels are highly correlated across samples. Subsequent annotation of modules often reveals biological functions and/or evidence of cellular specificity for cell types implicated in the tissue being studied. There are multiple ways to perform such analyses with weighted gene co-expression network analysis (WGCNA) amongst one of the most widely used R packages. While managing a few network models can be done manually, it is often more advantageous to study a wider set of models derived from multiple independently generated transcriptomic data sets (e.g., multiple networks built from many transcriptomic sources). However, there is no software tool available that allows this to be easily achieved. Furthermore, the visual nature of co-expression networks in combination with the coding skills required to explore networks, makes the construction of a web-based platform for their management highly desirable. Here, we present the CoExp Web application, a user-friendly online tool that allows the exploitation of the full collection of 109 co-expression networks provided by the CoExpNets suite of R packages. We describe the usage of CoExp, including its contents and the functionality available through the family of CoExpNets packages. All the tools presented, including the web front- and back-ends are available for the research community so any research group can build its own suite of networks and make them accessible through their own CoExp Web application. Therefore, this paper is of interest to both researchers wishing to annotate their genes of interest across different brain network models and specialists interested in the creation of GCNs looking for a tool to appropriately manage, use, publish, and share their networks in a consistent and productive manner.

## Introduction

Gene co-expression network analysis has been widely used to identify biologically important patterns in gene expression in a hypothesis-free and genome-wide manner (Miller et al., 2010; Forabosco et al., 2013; Uk Brain Expression Consortium (UKBEC), et al., 2016; de la Torre-Ubieta et al., 2018; Schizophrenia Working Group of the Psychiatric Genomics Consortium, et al, 2018; Bettencourt et al., 2019; Mencacci et al., 2020). The driving principle behind co-expression network analysis is that genes with highly correlated expression levels are also likely to share functional and biological relationships (Bakhtiarizadeh et al., 2018; Ma et al., 2018). With this in mind, gene co-expression networks (GCNs) can be viewed as models of how genes cluster together into modules of highly co-expressed genes through the use of graph-based approaches (Wolfe et al., 2005; Margolin et al., 2006; Langfelder and Horvath, 2008; Botía et al., 2017). Starting from an expression profile generated on a set of samples, the clustering process generates mutually exclusive gene sets (i.e., gene clusters). The resulting clusters are then annotated through a process which aims to describe them functionally and by their cellular specificity, amongst other properties. Using this approach, we can get a reasonably accurate summary of the input samples at the mRNA level. These models have value in themselves, as a means of efficiently describing the source gene expression profile. However, they can also be used to annotate external gene sets (Forabosco et al., 2013; Uk Brain Expression Consortium (UKBEC), et al., 2016; Salpietro et al., 2017; Bettencourt et al., 2019; Efthymiou et al., 2019;) generated under different experimental settings and conditions. The vast majority of analyses in the literature using GCNs as an analytic tool, focus on isolated GCNs created from specific sample sets under specific conditions. However, GCNs are more powerful when we considered collectively. For example, if we want to study neuro-degenerative diseases at the gene level, and how specific genes behave in terms of their co-expression, it is much more useful to study the gene set of interest across different brain regions (i.e., those particularly vulnerable to disease, in comparison with those which are less or never affected), and in unrelated tissues (e.g., skin). With this in mind, it is tremendously useful to have a tool which enables gene sets to be studied across all conditions in a comparative manner, including predictions based on module membership about the genes’ functions and cellular specificity across the conditions of interest.

Gene annotation is a basic task in bioinformatics. Whatever the process that led to identification of a gene set of interest (e.g., differential expression analysis, cellular screens, GWAS analysis or new gene discovery), a posterior annotation process is required. Thanks to the availability of manually curated databases of biological terms and their associated genes like the Gene Ontology (The Gene Ontology Consortium, 2017), it is possible to accurately annotate sets of genes with their predicted function (Conesa et al., 2005; Binns et al., 2009; Carbon et al., 2009; Eden et al., 2009; Supek et al., 2011). DAVID (Huang et al., 2009) and GSEA (Subramanian et al., 2005) represent examples of two different types of tools for gene annotation based on the available ontologies. DAVID and similar tools identify ontology terms which are enriched in the gene set of interest. Whereas, GSEA looks for significant overlaps between the gene set of interest and the gene sets found in MSigDB (Subramanian et al., 2005; Liberzon et al., 2015). This is a collection of manually curated and previously annotated gene sets organised under a variety of criteria. GSEA annotates genes based on how they are expressed across a phenotype but also on how they cluster together across all gene sets belonging to MSigDB. While the combination of GSEA and MSigDB form a general-purpose tool, CoExp is focused on providing gene sets that emerge from co-expression models. In CoExp, gene sets are grouped into a tissue-based hierarchy (e.g., gene sets discovered in neuropathologically normal putamen samples or gene sets discovered in frontal cortex samples originating from individual’s with Alzheimer’s disease). CoExp can show a user whether their genes of interest cluster in a significant manner within a given condition (see Supplementary Table 1 for a list of available networks), the functional characterization of the gene cluster and whether it is enriched for any specific cell type.

**FIGURE 1:**
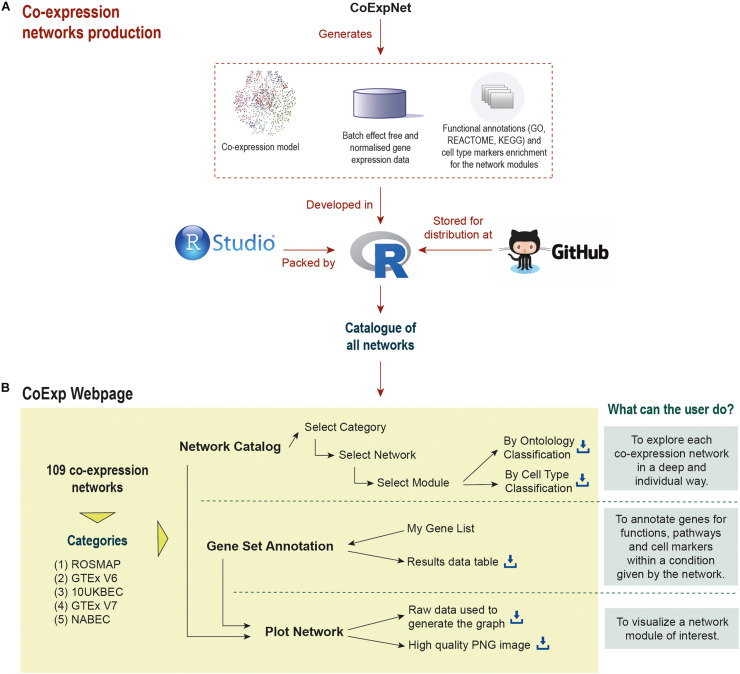
Life cycle of a co-expression model in CoExp: **(A)** the network is built from an expression profiling data set using the CoExpNets package. This generates a network, a set of residuals as a result of data QC processes and annotations for gene module function and cellular specificity. All networks are integrated into R packages (with a package for each category, namely ROSMAP, GTEx V6 and V7, UKBEC, and NABEC). These are made available within GitHub (see the links at the “Availability and Implementation” section of this paper). The packages are then integrated into the Web application **(B)**. There they can be exploited and visualised.

All GCNs within CoExp have been created using a similar pipeline based on Weighted Gene Co-expression Network Analysis [WGCNA (Langfelder and Horvath, 2008)], optimised with k-means (Botía et al., 2017) and annotated for function and cellular specificity (see Figure 1). A network model is a data table, with an entry for each gene, that can be shared and used in the form of a text file. Managing networks as text files is not easy because it is a manual task and therefore prone to error. Furthermore, to use large text files for gene set annotation requires coding skills so that module annotation can be performed efficiently across many different network models (i.e., different networks built using different expression data sets). Furthermore, R’s command-line environment reduces its usability in a world where the web-page format has become the most well-known and accepted way of browsing information. CoExp automates the annotation process and, most importantly visualisation of the underlying graph-based model on which co-expression networks are based, creating a more natural way to explore this type of data in a visual and interactive manner. Cytoscape (Shannon et al., 2003; Saito et al., 2012) is the pioneer stand-alone tool to provide generic network visualization, amongst many other functionalities including network generation. CoExp follows its approach, but tailored to GCNs.

In order to address these issues, we propose the CoExp web: a web-based application which aims to increase the usability and accessibility of co-expression network data. We illustrate the use of the CoExp web through the release of 109 different co-expression networks offered by the CoExpNets R package. We extend the functionality provided by the CoExpNets R package, through CoExp web’s “Plot Network” option, which generates a directed graph to visualise the most important genes from a preferred module. Therefore, CoExp is a convenient way to deploy, share, and use any co-expression models.

## Methods

### Co-expression Networks Generation

To construct co-expression models, we start from a gene expression profiling matrix E=M_sxg_ with samples listed as rows and genes as columns. Note that how the gene expression profiling was generated is not critical, i.e., we can either construct co-expression models from microarray or RNA-sequencing data. In the current CoExp GCN catalogue, there are both microarray and RNA-seq based GCNs. The co-expression pipelines process this matrix to obtain an adjacency matrix, A = M_gxg_ (see below) reflecting how adjacent to each other the genes are in terms of co-expression. *A* is then converted into a distance matrix D = M_gxg_, required for the clustering process. As a result of this process, we obtain gene groups in the form of a gene partition *P*

$$P=\left\{P_{i}\right\},1\leq i\leq kand\overset{k}{\underset{i=1}{⋃}}P_{i}=G,$$

such that the P_i_ are groups of genes, usually termed modules. The partition P can be disjunct, i.e., ⋂*P*_i_ = ∅ or we may have a global membership function we can use on any gene and partition, μ(g,P_i_) to generate values in [0,1] such that any gene may be a member of any partition to a certain degree. Disjunct partitions are easier to interpret and therefore they are more frequently used. In CoExp, genes belong only to a single group (i.e., module). In summary, given an expression profiling *E*, a GCN is a pair GCN(E) = (A, P), i.e., A is an adjacency matrix and P a partition.

Depending on the biological question we aim to study using GCNs, the *E* matrix can be treated differently. For example, we may try to correct *E* for any bias introduced by batch effects (Leek and Storey, 2007) or for any biological covariate whose probable effect will bias the models (e.g., sex or age). All the GCNs in CoExp have been corrected for batch [with the ComBat (Johnson et al., 2007) R package], for unknown latent effects with SVA and for gender and age (and post-mortem interval when this information is available) by regressing out the covariates through linear regression. In order to create all the GCNs, we first follow the standard WGCNA procedure: we identify the smoothing parameter that guarantees scale free topology for the network, we generate an adjacency matrix and the Topology Overlap Matrix (TOM). 1-TOM is used as the distance for hierarchical clustering. The gene clustering we obtain is subsequently refined using the k-means clustering algorithm (Botía et al., 2017). All networks include module membership for each gene (a measure of a gene’s relevance within the cluster) and the eigengenes (the 1st PCA of a module’s gene expression). Finally, the gene modules are annotated using gProfileR [see details here (Reimand et al., 2007)]. All networks are annotated for cellular specificity by performing a Fisher’s Exact test on the overlap between selected gene markers (found in the CoExpNets package) and genes within each module.

### The CoExp Web Software

**FIGURE 2:**
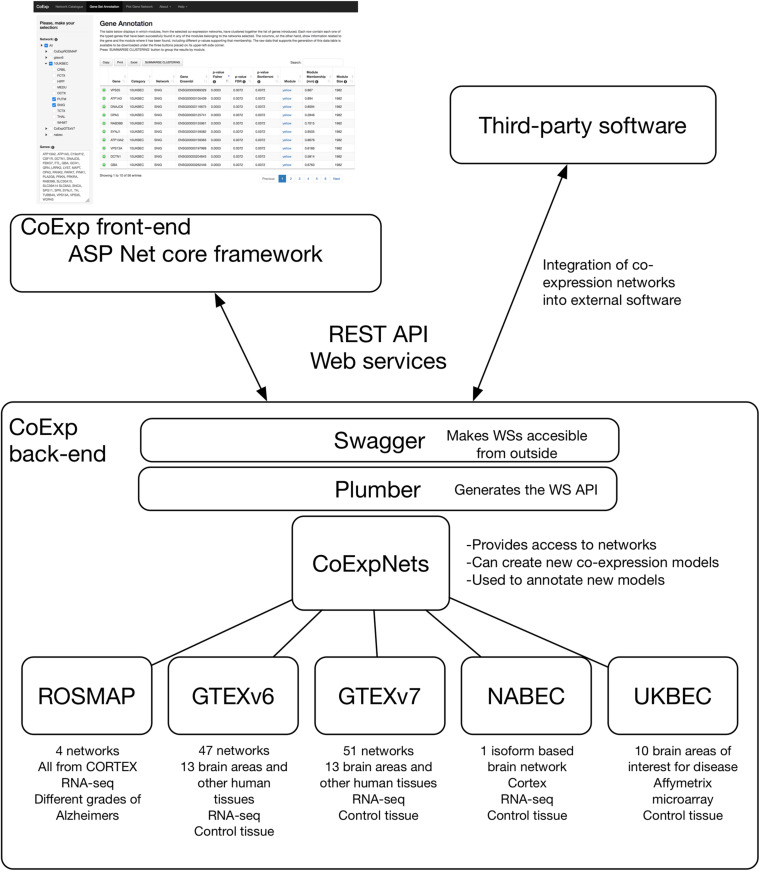
The software architecture of CoExp. The back-end includes the CoExpNets suite of packages. These models are made accessible through Web services using Plumber and Swagger. The front-end is developed in ASP.NET but all models are also accessible for the research community through the same Web services API.

The CoExp software architecture is depicted in Figure 2. The CoExp website has been implemented with the aim of connecting two different runtime environments, an R based and an ASP.NET based environment. The front end of CoExp has been developed following a Model View Controller (MVC) architecture implemented through the ASP.NET Core MVC framework, which is a cross-platform and open-source tool from the ASP.NET Core family of frameworks. Note that we have chosen the MVC architecture to enable division of the program logic into three main components: the model, the view and the controller. This facilitates the interpretation of the code by any external user interested in using or modifying it. This is essential for the maintainability of CoExp software over the coming years.

The back end of CoExp is based on a suite of five independent R packages corresponding to five network families (see section “Results”) plus the CoExpNets package which provides the necessary code to generate new GCNs and with a unified API to all networks. To make the collection of CoExp R methods accessible to the front end, a web Application Programming Interface (API) was chosen to define the interactions between the two runtime environments. To build the API, the R back end list of methods were first published using the Plumber R package and then made accessible using the Swagger (OpenAPI) language-agnostic specification to describe them in a user-friendly interface. The corresponding documentation can be accessed here:^1^. The external API was built using REST Web services.

Finally, the web server we use is one from the Apache HTTP Server Project. The communication between the CoExp ASP.NET Core MVC libraries, which are natively served by a Kestrel server, and the APACHE server was made by using a reverse-proxy service, which acted as an intermediate layer connecting both servers.

To reduce the complexity of this architectural design and to make it possible for a user to install and use CoExp locally, both the front and back end have been encapsulated within two different Docker containers and made available on DockerHub^2^.

### The Suite of Packages to Store and Manage Networks

All GCNs made available within CoExp are organised into R Packages. Currently, there is one R package for each of the five categories of networks (see section “Results” and Figure 2). Together, they make the CoExpNets suite of packages. When a set of GCNs is created, they are encapsulated into a package in a predefined manner. Each package must include an R object with the network, and two CSV files with the functional and cell type enrichments, respectively.

In order to generate a new GCN, a gene expression profiling matrix is required with genes as columns and samples as rows. Columns must be named with the corresponding gene IDs and rows with the sample IDs. To create the GCN, we use the getDownstreamNetwork() R function at the CoExpNets package. This produces, as output, three files, including (1) a R object file (RDS type) with the network, (2) a csv file with the gProfileR output coming from annotation of all network modules with GO, REACTOME and KEGG terms, and (3) another csv file with the cell type enrichment signals. An additional and optional file, is the one including covariates of interest for the samples (e.g., age, sex, etc.). Therefore, a network component within a CoExp network suite R package is composed of these three files plus an additional file with the gene expression profile as it was used within the getDownstreamNetwork() call.

Let us suppose we have created a number of new GCNs using the procedure above. The next step toward their integration into CoExp includes packing all of them into an R package. We recommend RStudio software to manage the package creation, and GitHub as the repository for making it available. We have followed the following reference (Wickham, 2015) to create all GCN suites currently available at CoExp. All include a README file explaining the network contents of the package, an R folder for the R code, a man folder for the documentation of the functions to access the networks and an additional “i*nst*” folder with the files which together comprise each network. The R code accompanying each GCN R package must include an initDb() function which, when called installs the required file names in memory so CoExp knows which networks are available in each category and where to find each network file set when required. Two additional functions include getCovariates(), to obtain the sample covariates for each network, and generateModuleTOMs(), to create the matrix of distances between genes required to plot each network module.

All CGNs in all suites were created using gene expression profiles which were validated in their respective research projects. Moreover, only GCNs of high quality are included in all suites, i.e., they must include expressed genes above a threshold, they were checked for sample outliers, and all GCNs show abundant functional and cell type annotation across their modules.

## Results

The CoExp Web page consists of three separate tabs, corresponding to the three different ways of using the network models: (1) network catalogue browsing, (2) network-based annotation of gene sets, and (3) network module visualization through active graph plots.

All three tabs support the exploitation of the same collection of networks. This collection consists of 109 different co-expression networks (Supplementary Table 1) that belong to four different network groups: (1) the Religious Orders Study and Memory and Aging Project (ROSMAP) (Bennett et al., 2012a, b; De Jager et al., 2018) composed of four co-expression networks derived from post-mortem human frontal cortex originating from control individuals, as well as those with cognitive impairment and Alzheimer’s disease; (2) The Genotype-Tissue Expression project (GTEx) V6 and V7 (The GTEx Consortium, 2015) composed of two suites of GCNs on 47 and 51 post-mortem control human tissue samples, respectively; (3) United Kingdom Brain Expression Consortium (UKBEC) (Forabosco et al., 2013; UK Brain Expression Consortium, et al, 2014) composed of 10 microarray-based gene expression profiling networks derived from post-mortem control human brain tissue; (4) North America Brain Expression Consortium (NABEC) (Dillman et al., 2017), composed of one gene co-expression network derived from post-mortem control human frontal cortex. Through CoExp, we and others have used these models to provide annotations for genes and gene sets in a variety of papers (Chelban et al., 2017, 2019; Salpietro et al., 2017, 2018; Efthymiou et al., 2019).

Many GCNs currently available within CoExp are brain-related. ROSMAP, UKBEC, and NABEC are all brain-specific GCN sets. The GTEx packages also include GCNs for 13 different brain areas. This GCN set also includes GCNs for a wide variety of human tissues. This makes it possible to compare gene sets across brain regions, but also to identify brain-specific phenomena (those not seen in alternative tissues). Furthermore, GTEx networks, enable investigation of gene sets outside of brain.

### Network Catalogue Browser

The user can become familiar with the GCNs available by navigating through the catalogue. With this in mind, the first tab available on the upper menu corresponds to the “Network Catalogue” tab through which the user can inspect and download the whole network catalogue to obtain information about any network or any module within a network. To browse the catalogue, the first step consists of selecting a network category in the menu placed at the left-hand side of the webpage. The second step is the selection of a co-expression network of interest within that category. Finally, the user can select one of two different views: the ‘Ontology Classification’ or the “Cell Type Classification.” The “Ontology Classification” view returns a data table in which each module from the selected network occupies one row. The columns provide summarized information about annotation terms enriched for the genes in the modules. The p-value column shows the enrichment obtained from gProfileR (Reimand et al., 2007), which incorporates data from well recognised ontologies, including Gene Ontology, REACTOME, and KEGG. The “Cell Type Classification” view, returns a data table in which the rows correspond to sets of gene markers relevant to specific brain cell types tested for enrichment (Fisher’s Exact test) and each module occupies a column. Each cell within the table contains the Bonferroni corrected p-value for the enrichment of a set of cell type markers within a module. It is necessary to Bonferroni-correct (Benjamini and Hochberg, 1995) the Fisher’s exact p-values for multiple testing, as each module is tested against all cell type marker sets. In all cases, the data table can be downloaded as an excel file, using the “Excel” button placed on the upper-left-side corner of the table. Note that it is possible to regenerate all these annotations for each network by using CoExpNets::annotate() function on the desired GCN, locally.

**FIGURE 3:**
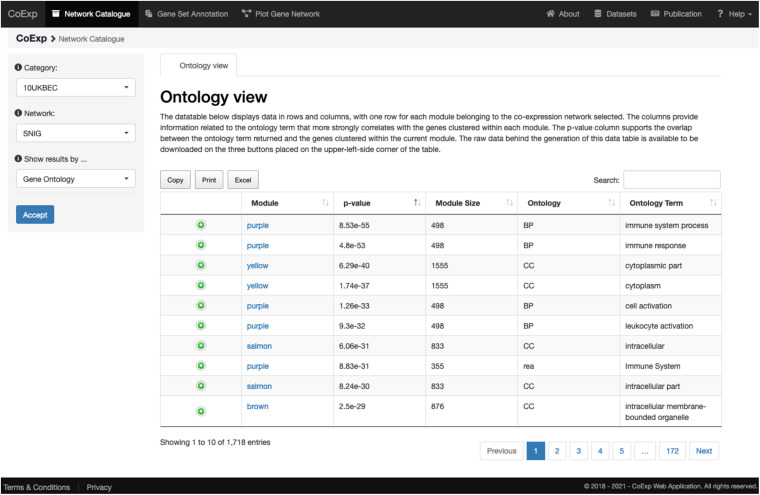
View of the network catalogue when we select the substantia nigra (SNIG) network from the 10UKBEC network family and use the Ontology View. It shows the table corresponding to the functional enrichments obtained using gProfileR. Enriched terms are ordered by p-value by default. In this case, the first two terms provide evidence that the genes in the purple module are enriched for immune-related functions. All annotations can be downloaded in the form of an Excel file for later inspection. Furthermore, if the user is interested in a particular annotation term, he/she can use the search text frame at the top right, to look for terms matching specific keywords. A visual demonstration of all these functionalities is available, as a video, at the Help section of the CoExp web under the “Network Catalogue” section.

**FIGURE 4:**
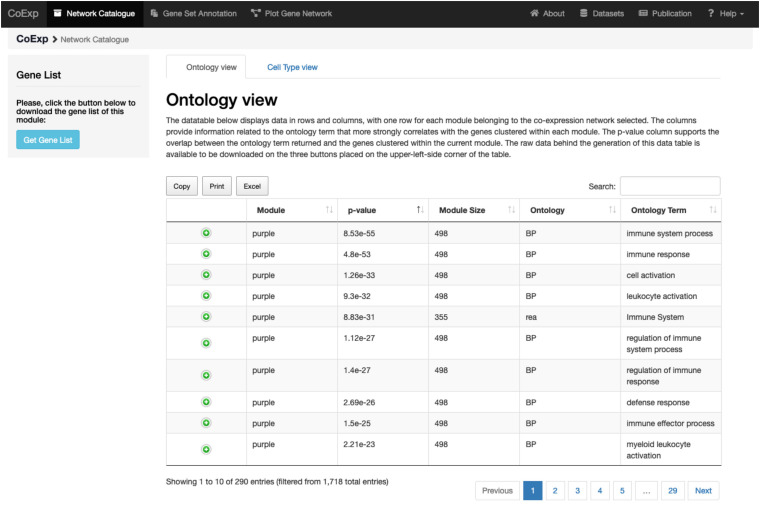
Investigating a module of interest. By clicking on a module of interest, such as the purple module link, the user obtains a table with all the purple module-specific information on functional annotation. All annotations can be downloaded in the form of an Excel file for later inspection.

We can illustrate its use with an example. After clicking at the tab, let us select, for example, the 10UKBEC GCN category and then the SNIG (substantia nigra) GCN. After clicking “Accept,” the “Gene Ontology” view returns a data table with a summary of the network clusters (Figure 3). It is notable that the “purple” module contains 498 genes expressed within UKBEC substantia nigra tissue, which are enriched for immune-related GO terms amongst others (Figure 4). This enrichment is unlikely to have occurred by chance based on the significant Bonferroni-corrected p-value of 8.55e-55 for the “immune system process” GO term. Similarly, after selecting the “Cell Type” view, we can see that the 498 genes within the “purple” module are enriched for microglial gene markers (p-value = 2.06e-91). Thus, navigating through only a couple of web interfaces we can explore any network module. Note that the Help section of the CoExp web has available a video illustrating how to use the Network catalogue browser under the section with the same name.

### Gene Set Annotation

GCNs are often used to annotate a gene set of interest, in the context of a specific condition (e.g., a tissue of interest). “Gene Set Annotation” is the second tab within the main menu. Using this function, the user can investigate whether his/her own gene set of interest is enriched within a single or multiple co-expression modules across all the co-expression networks from amongst those available in the catalogue. In this way, those genes can be annotated based on how they are distributed across the network modules and their biological context can be easily explored. If a gene of interest has not been found in any module a pop-up view will inform the user. The user is supplied a results table in which each row relates to a gene of interest which has been successfully found in any of the modules belonging to the network or networks selected. The columns provide information on the module in which the gene has been identified, including the statistical significance of the overlap between the input genes and the genes int the module, as well as a brief description of the module’s function based on the top five most-significantly enriched GO terms. All the outputs associated with this type of analysis are available for download using the three buttons placed on the upper-left- corner of the table. Furthermore, the Gene Set Annotation tab has default values for all the choices the user has to make before proceeding with the annotation task.

**FIGURE 5:**
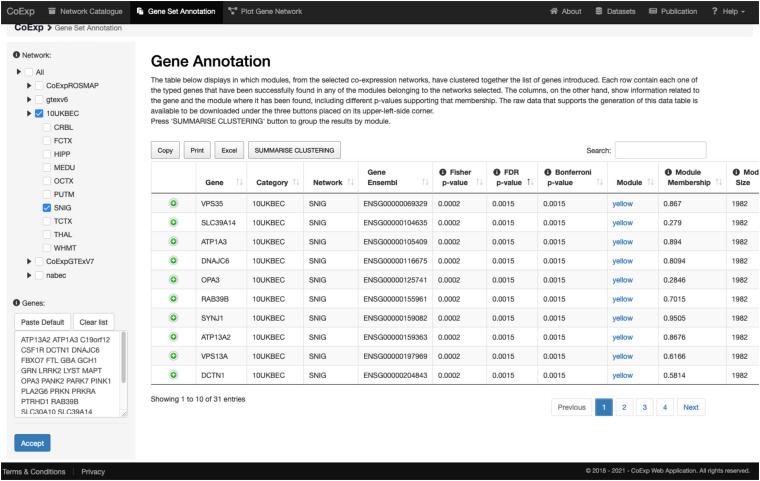
Use of the gene set annotation tab. This screen capture shows the results of using the Gene Set Annotation tab with 32 genes associated with juvenile Parkinson’s disease (visible within the text pane in the bottom left) and used as the default example when arriving at CoExp for the 1st time, together with default family and network values too (namely the SNIG network from UKBEC as used in Figures 3, 4). The analysis yields the results on the right table. Each row corresponds to information about one gene out of the 32 genes used for this example. For example, in the 1st row we see that *VPS35* has a module membership of 0.867 within the SNIG yellow module. Furthermore, *VPS35* significantly clusters within the yellow module with many other juvenile Parkinson’s disease genes. In fact, 10 out of the 32 genes cluster together within the yellow module. The module has 1,982 genes and the Fisher’s exact test for the overlap yields a significant p-value (FDR 5% *P* < 0.0072). If we click on the “yellow” link then we can inspect this module as we did for the purple module described in Figure 3, 4. In the case of the latter, we would see that that the module is enriched for multiple dopaminergic gene markers (*P* < 2.78 × 10^− 6^) and that the most significantly enriched GO terms include transport (*P* < 1.25 × 10^− 19^) and the establishment of localization (*P* < 3.16 × 10^− 18^) amongst others. All annotations can be downloaded in the form of an Excel file for further inspection. For a visual demonstration of this same example, the interested user may access a video-tutorial available at the Help section of the CoExp web under the “Gene Set Annotation” section.

Let us suppose we want to annotate 32 genes associated with Parkinson and complex parkinsonism as defined by Genomics England’s PanelApp (Martin et al., 2019), and we want to study this gene set in a biologically relevant GCN such as the substantia nigra network (SNIG of the 10UKBEC network family). This would involve: (1) deciding which GCN or GCNs will be of interest, (2) using CoExp to see whether there are potentially interesting gene clusters (i.e., a subset of our genes cluster together in specific modules within the GCNs selected), (3) use the Network catalogue for a better characterization of the genes, and (4) generate network plots of the genes of interest. In order to select potential GCNs of interest, the user can inspect the network catalogue through the “Network Catalogue Tab” as we have shown in the last subsection. The step in which we obtain how genes cluster across the networks is exemplified in Figure 5. It demonstrates that a large proportion of the 32 genes of interest cluster together in the yellow module of the SNIG network. The next step in the analysis would be to obtain more details about the yellow module by selecting it and so navigating back to the Network Catalogue where we can access full details of the module including its functional annotation and enrichment for cellular specificity (Figures 3, 4). Finally, how we obtain network plots is detailed in the following subsection. A video illustrating how to use CoExp with this particular example is also available at the CoExp Help page, under the “Gene Set Annotation” section.

### Plot Network

**FIGURE 6:**
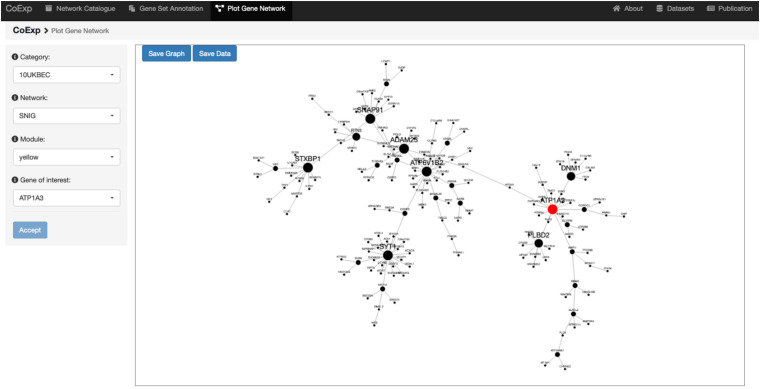
Use of the Plot Gene Network Tab. This figure shows the view we get when we use the Plot Gene Network tab to visualize the gene *ATP1A3*. We chose this gene because it is associated with PD and is one of the genes with high module membership within the yellow module of the SNIG GCN within the 10UKBEC category (Figure 5). After clicking the “Accept” button, the plot shows, highlighted in red, the gene *ATP1A3* as a very important gene within its module (as indicated by the large node size). There are 16 other genes, which appear as its nearest neighbour and they can be used to further study the transcriptomic context of *ATP1A3*. Note that this example has been visually recreated in a demonstration video available at the Help section of the CoExp web under the “Plot Gene Network” section.

Once the user has decided which network is of interest, and which module within the network requires detailed visualization, the genes can be plotted. The third tab, “Plot Network,” enables the graph-based visualisation of the genes within a module of interest, whether identified by browsing through the catalogue or because the user’s gene set of interest significantly clusters within that module. The “Plot Network” tab generates an interactive directed graph formed by the hub genes within a module. The user can select how many of the most relevant genes will appear in the plot. The resulting plot is interactive in the sense that it can be zoomed, rotated, and the direct neighbours of any gene highlighted by just clicking on the gene of interest. Both the raw data and a high quality PNG image of the graph are available for download. See Figure 6 for the result of selecting the ATP1A3 gene as the main gene of the network plot we want to obtain, for the running example of the 32 PD genes analysis on the SNIG 10UKBEC GCN. See also the available video for this very same example available at the CoExp web help page, under the “Plot Gene Network” section.

## Discussion

CoExp web is a web platform that enables the exploitation of co-expression networks. CoExp currently offers 109 co-expression models focused on brain transcriptomics with plans to expand its scope. It is a powerful, easy-to-use, and innovative tool for gene set annotation across a variety of brain-specific transcriptomic data sets, including also a variety of non-brain tissues that may be used as controls or on their own. CoExp makes co-expression models visually manageable, accessible, and easily exploitable by the scientific community. Everything is shareable in CoExp: all GCNs, the expression profiles from which they were created and their annotations are accessible within GitHub (see the links at the “Availability and Implementation” section of this paper). Furthermore, both the back and front-end software from which the CoExp Web application is generated are readily accessible such that any research laboratory can construct its own CoExp web site. This makes it a powerful tool for the wider research community interested in producing, using or sharing GCNs to support their research.

### Future Works

We are extending the scope of CoExp in a number of areas. Firstly, we intend to expand the number of available GCNs. This will include incorporating GCNs generated using additional brain-related bulk RNA-sequencing data from projects, such as CommonMind (Hoffman et al., 2019) and PsyhcEncode (Wang et al., 2018). We aim to integrate them into CoExp as CGN suites. We are also working toward the generation of GCNs based on single-cell/single-nucleus transcriptomic datasets, including the single-nucleus RNA-sequencing data released by ROSMAP.

A second area of work is the integration of available sample covariates into the CGNs. These are very important to annotate models, as for example when a user wants to identify network modules which correlate with age of the samples, or with the case/control condition to identify disease-related modules. This will have an impact on the CoExp functions and Web interface.

A third expansion area is the inclusion of GCNs created by collaborators or any other members of the research community interested in publishing their networks. We are working on defining a CoExp network generation pipeline to satisfy a minimal level of quality for the CGN to be acceptable for publication at our Web. This particular area will also require a Web facility for automated network submission through the Web. In the meantime, we are happy to accept contributions in the form of new GCNs to be added to the CoExp catalogue. Any researcher willing to contribute to CoExp by submitting their own GCNs may contact the corresponding author and we will provide the necessary guidance.

Fourthly, the current implementation of CoExp runs on a single R environment at the back-end. This has direct impact on the number of concurrent users it can support. Concurrent requests to CoExp will be queued and attended to sequentially. We are currently working on enabling a multi-user CoExp environment thorough multiple docker images served through an HTTP proxy.

Finally, we plan to improve CoExp usability. To date, CoExp usability testing was conducted through the definition of use cases, represented as storyboards with each vignette designed to visualise input actions and the output of each interaction between the user and the Web application. These were later used at two hackathon meetings with specialised users from an international genomics consortium (the International Parkinson Disease Genetics Consortium), where there was an opportunity to work through sample questions to illustrate CoExp usage and to define possible analyses. We used the very valuable feedback gathered from the meetings to improve the CoExp user interface. Our future plans include the organisation of more hackathons not only to improve CoExp usability, but also to expand its community of users.

## Data Availability Statement

The datasets presented in this study can be found in online repositories. The names of the repository/repositories and accession number(s) can be found below: https://rytenlab.com/coexp.

## Author Contributions

SG-R developed the CoExp Web tool. AG-M, AC, FJ-R, and RR participated in the creation of the gene co-expression networks and CoExpNets suite of packages. MC supervised the NABEC co-expression generation from scratch. JH participated in the project design. JB supervised the creation of the CoExpNets family of packages. MR and JB co-directed the whole project. All authors participated in the paper writing up and all critically reviewed the manuscript.

## Conflict of Interest

The authors declare that the research was conducted in the absence of any commercial or financial relationships that could be construed as a potential conflict of interest.

## Footnotes

1 https://rytenlab.com/swagger/index.html

2 https://hub.docker.com/r/soniaruiz/coexp

## Availability and Implementation

The CoExp Web is accessible at https://snca.atica.um.es/coexp/.

The back and front ends code of CoExp Web application is fully available for download at GitHub at https://github.com/SoniaRuiz/CoExp_Web.

The docker images of CoExp web are available for download on Docker Hub at https://hub.docker.com/r/soniaruiz/coexp.

The CoExpNets suite of packages can be accessed in the following links

http://github.com/juanbot/CoExpNets

http://github.com/juanbot/CoExpROSMAP

http://github.com/juanbot/CoExp10UKBEC

http://github.com/juanbot/CoExpGTEx

https://github.com/juanbot/CoExpGTExV7

http://github.com/juanbot/CoExpNABEC

Contact: juanbot@um.es
